# Comparison of two different intravitreal treatment regimens combined with systemic antiviral therapy for cytomegalovirus retinitis in patients with AIDS

**DOI:** 10.1186/s12981-023-00543-x

**Published:** 2023-07-14

**Authors:** Xuemei Liang, Hongmei An, Huawei He, Baiyun Shen, Zuguo Ou, Li Li

**Affiliations:** 1 Department of Fundus Disease, Nanning Aier Eye Hospital, Nanning City, China; 2 Department of Infectious Diseases, The Fourth Hospital of Nanning, Nanning City, China

**Keywords:** Acquired Immune Deficiency Syndrome, Cytomegalovirus Retinitis, Intravitreal Injection, Visual outcome, Injection frequency

## Abstract

**Purpose:**

To compare the efficacy and injection frequency of intravitreal low-dose vs. intermediate-dose ganciclovir therapy in acquired immune deficiency syndrome (AIDS) patients exhibiting cytomegalovirus retinitis (CMVR).

**Methods:**

A prospective, single-centre, double-blinded, randomized controlled interventional study was conducted. Fifty patients with a total of 67 included eyes were randomly divided into low-dose (0.4 mg ganciclovir per week) and intermediate-dose (1.0 mg ganciclovir per week) groups. The primary clinical outcomes were the changes in best corrected visual acuity (BCVA) from baseline to the end of treatment and the 12-month follow-up visit as well as the number of intravitreal injections.

**Results:**

In both groups, the median BCVA, expressed as the logarithm of the minimum angle of resolution (logMAR), improved significantly from baseline to the end of treatment (both *p* < 0.001), while vision loss from CMVR continued to occur at the 12-month visit. The mean number of injections was 5.8 in the low-dose group and 5.4 in the intermediate-dose group. No significant differences were detected between the two groups (*p* > 0.05). Regarding the location of CMVR, we found that Zone I lesions led to a worse visual outcome, more injections and a higher occurrence rate of complications than lesions in other zones (*p* < 0.05).

**Conclusions:**

The efficacy and frequency of injections to treat CMVR in AIDS patients were not significantly different between low and intermediate doses. Zone I lesions were associated with a worse visual outcome, more injections and a higher occurrence rate of CMVR-related complications than lesions in other zones.

## Background

Human immunodeficiency virus (HIV) is a retrovirus that replicates in CD4 T lymphocytes and causes a multisystem disease called acquired immune deficiency syndrome (AIDS) [1]. Cytomegalovirus retinitis (CMVR) is the most common opportunistic ocular infection and the most serious cause of visual loss in advanced AIDS; it occurs primarily among those with CD4^+^ T-lymphocyte counts < 50 cells/µL [2]. Antiretroviral therapy (ART), an established treatment, has reduced the incidence of CMVR by 80% [3]; nonetheless, 25–42% of AIDS patients develop CMVR [4]. The rates of visual impairment (visual acuity worse than 20/40) and blindness (visual acuity 20/200 or worse) are 0.9/100 and 0.4/100 person years, respectively, if CMVR is not diagnosed and treated as early as possible [5].

Ganciclovir has been approved for administration via two routes to treat CMVR: intravenous infusion and intravitreal injection. Intravitreal injection is an easy, safe, and well-tolerated means of administration that effectively increases retinal tissue concentrations without triggering systemic toxicity [6]. A variety of ganciclovir doses, ranging from low (0.2–0.4 mg/0.1 mL) to high (2–6 mg/0.1 mL), have been reported to be clinically effective [7–9]. However, an animal experimental study showed that after intravitreal injection (IVI) of 3 different doses of ganciclovir, retinal morphology and function remained normal in the 400 µg group, whereas retinal toxicity was found in the 2 mg and 5 mg groups; the latter showed especially severe toxicity [10]. Choopong et al. [11] reported a case of crystal formation in the vitreous cavity after IVI of ganciclovir (4 mg/0.04 mL), which resulted in optic atrophy and loss of visual acuity. At present, there is still no standard regimen of intravitreal ganciclovir injection for CMVR treatment. With regard to the injection intervals, the recommended therapy schedule is twice weekly during a three-week induction period, followed by maintenance therapy once a week until the lesion becomes a scar [9]. In one report, patients received injections at weekly intervals [12]. In addition, pharmacokinetic studies have demonstrated that the concentrations of ganciclovir in the vitreous after one injection can be maintained above the median effective concentration for up to one week [13]. In addition, we previously observed good clinical therapeutic effects of weekly IVI of 0.4 mg ganciclovir in AIDS patients presenting CMVR, without clinical signs of retinal or optic nerve toxicity [14]. Based on our previous clinical experience and the literature, it seems that low-dose ganciclovir requires more injections. However, few studies have been published on the injection frequency of different intravitreal ganciclovir dosages for CMVR.

Therefore, in our study, all patients received intravenous ganciclovir for 2 weeks, combined with.

IVI of ganciclovir at a dose of 0.4 mg or 1.0 mg. We consider this regimen a suitable balance of therapeutic effectiveness, retinal toxicity, and burden for low- to middle-income patients in Southwest China. We thus undertook this investigation to compare the efficacy and injection frequency of 0.4 mg vs. 1.0 mg ganciclovir for AIDS patients with CMVR.

## Methods

### Study design

This was a prospective, single-centre, double-blinded, randomized controlled interventional study. Sixty-seven eyes of 50 AIDS patients with CMVR were enrolled in this study. All HIV patients received ART and systemic antiviral therapy at The Fourth Hospital of Nanning, and AIDS-related CMVR was treated at Nanning Aier Eye Hospital.

### Participant recruitment

Fifty patients were randomly divided into a low-dose group (ganciclovir 0.4 mg/0.1 mL per week, n = 25) and an intermediate-dose group (ganciclovir 1.0 mg/0.1 mL per week, n = 25) after diagnosis with CMVR. These random allocations were concealed from the subjects and the investigators to prevent bias. The inclusion criteria were that patients were first diagnosed with AIDS-related CMVR and were at least 18 years old. CMVR diagnosis was based on wide-angle fundus photographs showing the characteristic retinal changes. These changes comprise minimal to moderate anterior and vitreous inflammation, retinal necrosis with haemorrhages and retinal vasculitis with yellow–white lesions, also known as “pizza pie retinopathy” or “cottage cheese with ketchup retinopathy” [1]. Additionally, all patients underwent intraocular viral nucleic acid testing (including CMV, herpes simplex virus (HSV), varicella zoster virus (VZV), Epstein–Barr virus (EBV), and human herpesvirus (HHV)-6) by real-time fluorescent quantitative polymerase chain reaction (qPCR) to make a definite differential diagnosis. The exclusion criteria were as follows: 1) patients previously treated with intravitreal injection anti-CMV treatment; 2) patients with retinal detachment or no light perception; and 3) patients with glaucoma, diabetes mellitus, cataracts or other AIDS-related diseases that affect vision.

### Treatment

All HIV patients received ART. After being diagnosed with AIDS-related CMVR, patients were encouraged to receive systemic ganciclovir 5 mg/kg q12h for 2 weeks by intravenous drip and then continued anti-CMV therapy by oral administration of 1 g ganciclovir 3 times per day. The decision to stop systemic ganciclovir was usually based on stable CD4^+^ cell counts > 200 cells/µL lasting for at least 3 months or a decrease in the neutrophil count to 1.0 × 10^9^/L. For weekly IVI, patients were first anaesthetized with topical proparacaine. Then, 0.4 mg or 1.0 mg ganciclovir (Hunan Wuzhou tong Pharmaceutical Co., Ltd, Changsha, China) was injected with a 30-gauge needle placed in the vitreous cavity through the pars plana in the superonasal or superotemporal quadrant of the eye. Before the initial injection, 50 µL of aqueous humour was extracted by paracentesis at the 5 o’clock position, and the aqueous humour sample was sent to Beijing Giantmed Medical Diagnostics Laboratory for intraocular virus nucleic acid testing. Fundus examination was performed before each reinjection and once a month during the follow-up period. The treatment endpoint was defined as the disappearance of yellow–white lesions and gradual retinal scarring, which were observed by two senior doctors (X.M.L. and B.Y.S.).

### Clinical data

The clinical data of all eligible patients were recorded. Baseline data, including age, sex, right or left eye involvement, duration of HIV, duration of eye symptoms, CMV DNA load in the aqueous humour, and serum CD4^+^ T-lymphocyte counts, were collected. Ophthalmic data included best corrected visual acuity (BCVA), intraocular pressure (IOP), location of CMVR at the time of diagnosis, number of injections, and rates of CMVR-related complications. BCVA measurements were made using decimal charts and then converted to the logarithm of the minimum angle of resolution (logMAR) for reporting purposes. For patients who could not discern any lines of the chart, a score of 2.0 logMAR units was assigned for counting fingers; 2.3 logMAR units, for detecting hand motion; 2.7 logMAR units, for light perception; and 3.0 logMAR units, for no light perception (NLP) [15]. The fundus lesions were divided into three zones. Zone I included the area within 1500 μm of the nerve or 3000 μm of the fovea; Zone II included the area outside Zone I and extending to the vortex veins; and Zone III included the area from the vortex veins to the ora serrata [16].

### Study outcomes

The primary clinical outcomes were the changes in BCVA from baseline to the end of IVI treatment and the 12-month visit, as well as the number of IVIs. The secondary outcome measures were the percentages of patients with improved, stable and decreased BCVA; the prevalence of visual impairment and blindness at 12 months; and the occurrence of CMVR-related complications and other adverse events. Visual impairment and blindness were defined as visual acuity worse than 20/40 and worse than or equal to 20/200, respectively. Therapeutic efficacy was divided into three levels: (1) improved: vision improved by ≥ 2 lines on the acuity chart; (2) stable: vision fluctuated by less than 2 lines; and (3) decreased: vision worsened by ≥ 2 lines on the acuity chart or progressed to NLP.

### Data analysis

All statistical analyses were performed using IBM SPSS Statistics version 22 (IBM Corp, Armonk, NY, USA). The normality of the data was tested by the Kolmogorov–Smirnov test, which showed that none of the variables except age followed a normal distribution. Categorical variables were compared using the chi-square test. Continuous variables were analysed using the Mann–Whitney U test. Changes in BCVA at various time points compared to baseline were tested using the Wilcoxon signed-rank test. Statistical significance was defined as *p* < 0.05. All tests were two-sided.

## Results

### Participant characteristics

A total of 67 eyes of 50 AIDS patients with CMVR (41 males and 9 females) were enrolled in the study. The median baseline CD4^+^ T-lymphocyte count was 24.7 ± 25.1 cells/mm^3^ (range, 1–177 cells/mm^3^). With the continuation of systemic and intravitreal antiviral therapy, the number of CD4^+^ T cells increased gradually. The CD4^+^ T-cell count was 411.6 ± 156.4 cells/mm^3^ (range, 189–809 cells/mm^3^) at the 12-month visit. Table 1 describes the baseline demographics and clinical features. Among all the affected eyes, 42 eyes (62.7%) were in Zone I, while the remaining eyes (37.3%) were in Zone II. Visual impairment and blindness were found in 12 eyes (17.9%) and 34 eyes (50.7%), respectively. The mean duration of eye symptoms was 40.1 ± 37.9 days. Age, sex, eyes, HIV duration, HIV virus load, HIV suppression rate (Viral load < 20 copies /mL or no virus detected), duration of eye symptoms, lesion location, baseline BCVA and IOP, and CMV DNA load were not significantly different between the two groups.

**Table 1:** Demographics of the participants and baseline characteristics of CMVR in this study

	Low-dose group (patients: n = 25, eyes: n = 36)	Intermediate-dose group (patients: n = 25, eyes: n = 31)	*p*
Age (years), mean ± SD (range)	43.4 ± 16.8 (20 ~ 67)	42.8 ± 17.3 (23 ~ 68)	0.929
Sex (male/female)	22/3	19/6	0.269
Eyes (bilateral/unilateral)	11/14	6/19	0.136
Duration of HIV, years Median ± IQR (range)	3.0 ± 2.5 (0.4 ~ 7)	3.0 ± 5.0 (0.2 ~ 9)	0.613
HIV suppression rate	61%	69%	0.38
Duration of eye symptoms, days, median ± IQR (range)	32.0 ± 46.5 (3 ~ 180)	30.0 ± 35.5 (5 ~ 180)	0.970
Intraocular pressure (mmHg), median ± IQR (range)	13.2 ± 4.5 (9.0 ~ 19.8)	14.1 ± 5.2 (8.5 ~ 20.0)	0.661
BCVA (logMAR), median ± IQR (range)	1.2 ± 1.4 (0.2 ~ 2.7)	0.9 ± 1.8 (0.1 ~ 2.7)	0.663
Lesion location (Zone I/Zone II)	25/11	17/14	0.317
CD4^+^ T-cell count (cells/mm^3^) median ± IQR (range)	22.5 ± 28.5 (1–177)	27.8 ± 22.1 (3–278)	0.996
HIV virus load (copies·mL^− 1^) Median ± IQR (range)	118 ± 2318.5(22–6793)	159 ± 1979.7 (25–8521)	0.509
Intraocular CMV DNA load (copies/mL) median ± IQR	(3.5 ± 2.0) ×10^4^	(4.3 ± 6.3) ×10^4^	0.649

SD, standard deviation; IQR, interquartile range

### Visual outcomes

Table 2 shows the BCVA change and final therapeutic efficacy in the low-dose and intermediate-dose groups. In the low-dose group, the baseline median BCVA in logMAR units was 1.2 (interquartile range (IQR) ± 1.4), which improved to 0.45 (IQR ± 1.18) at the treatment endpoint (*p* < 0.001) and decreased to 0.7 (IQR ± 1.15) at the 12-month visit (*p =* 0.126). In the intermediate-dose group, the baseline median BCVA in logMAR units was 0.9 (IQR ± 1.8), which improved to 0.5 (IQR ± 1.2) at the treatment endpoint (*p* = 0.001) and decreased to 0.7 (IQR ± 2.1) at the 12-month visit (*p =* 0.094). In short, all patients had a significant improvement in BCVA after treatment. However, BCVA decreased further at the 12-month visit, by which time it was not significantly higher than the baseline value. No significant differences were detected in the BCVA change or therapeutic efficacy between the two groups at any time point (*p* > 0.05). At the 12-month visit, 51.5% of all affected eyes had improved BCVA, 25.8% of eyes remained stable, and 23.9% of eyes had degraded BCVA. Regarding the location of the CMVR, 42, 25, and 0 eyes had lesions in Zone I, Zone II, and Zone III, respectively. At the 12-month visit, patients with Zone I involvement had a significantly worse visual outcome than those with Zone II involvement (1.4 ± 2.0 vs. 0.4 ± 0.2 logMAR units, *p* < 0.001). Visual impairment and blindness were observed in 28 eyes (41.8%) and 25 eyes (24.2%), respectively. Loss of vision occurred mainly due to direct macular involvement and CMVR-related complications, and 4 eyes progressed to NLP because of immune recovery uveitis (IRU) and neural atrophy.

**Table 2:** Summary of visual outcomes

	Low-dose group Eyes: n = 36	Intermediate-dose group Eyes: n = 31	*p*
Initial BCVA	1.2 ± 1.4(0.2 ~ 2.7)	0.9 ± 1.8(0.1 ~ 2.7)	0.663
Treatment-endpoint BCVA	0.45 ± 1.18	0.5 ± 1.2	0.558
Final BCVA	0.7 ± 1.15	0.7 ± 2.1	0.256
Final therapeutic efficacy			0.815
Improved	19/36 (52.8%)	15/31 (48.4%)	
Stable	8/36 (22.2%)	9/31 (29.0%)	
Decreased	9/36 (25.0%)	7/31 (22.6%)	

BCVA, best corrected visual acuity (logMAR visual acuity)

### Number of IVIs

The mean number of IVIs was 5.8 ± 1.6 (range 3–10) in the low-dose group and 5.4 ± 1.1 (range 4–8) in the intermediate-dose group (*p* = 0.347). In terms of the location of CMVR, 42 eyes with lesions in Zone I and 25 eyes with lesions in Zone II received an average of 5.9 ± 1.5 (range 3–10) and 5.2 ± 1.1 (range 4–8) injections, respectively (*p* = 0.036). These results suggested that eyes with Zone II lesions required significantly fewer IVIs than those with Zone I lesions.

### CMVR-related complications and other adverse events

**Fig. 1:**
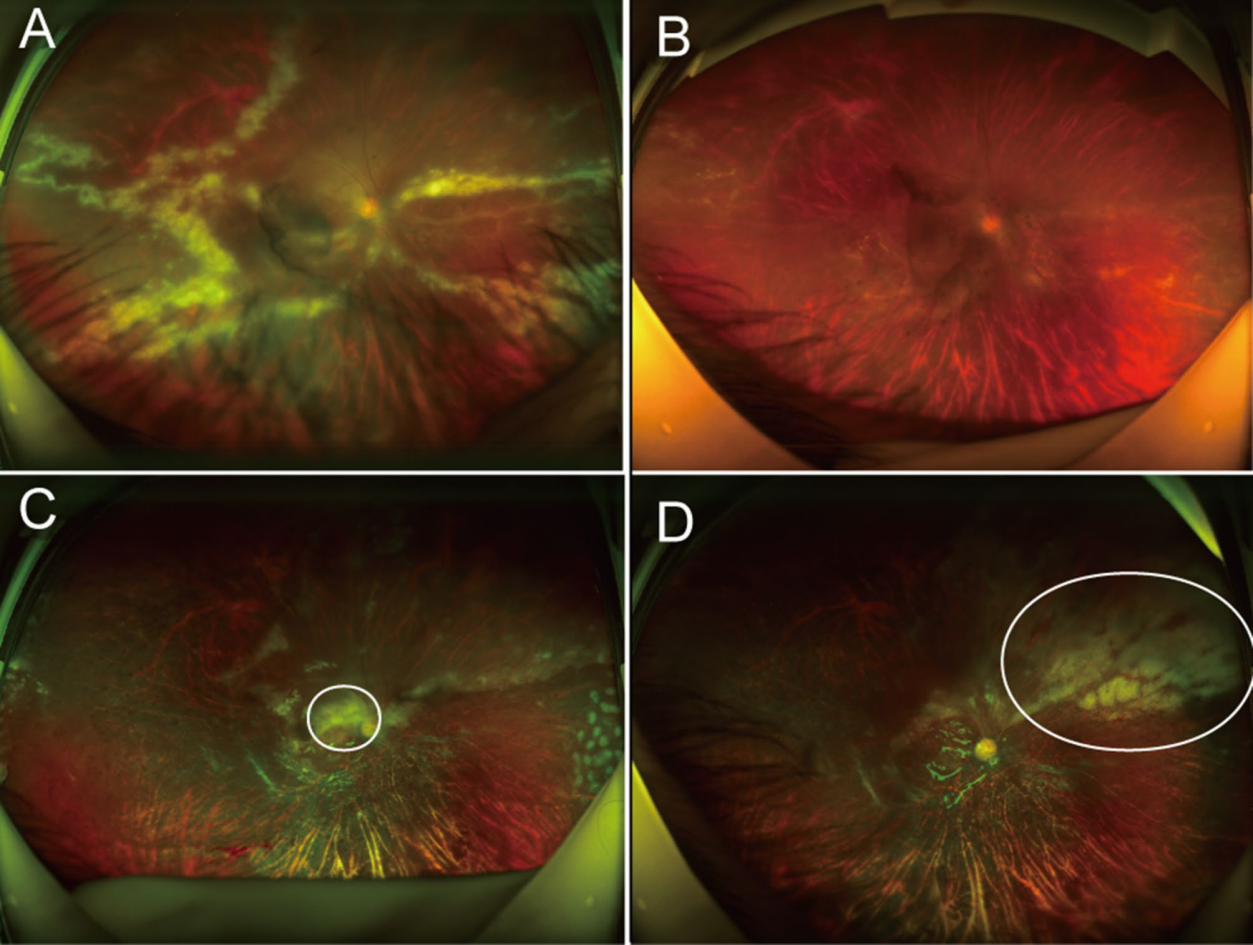
A CMVR lesion in Zone I was treated with injections of 0.4 mg ganciclovir. **A**: Before the treatment. **B**: Three weeks (3 injections) after treatment, the haemorrhage and yellowish-white exudate had clearly been absorbed. **C**: Eight months later, the patient developed rhegmatogenous retinal detachment and received 25-gauge pars plana vitrectomy combined with silicone oil tamponade. CMVR relapse occurred even after the vitreous cavity had filled with silicone oil (new exudation shown in the circle). **D**: Two months after atrophy of the macular lesion, a yellowish-white exudate appeared again on the nasal side (shown in the circle)

Complications occurred in 35 eyes (52.2%; Zone I in 26 eyes and Zone II in 8 eyes) during the follow-up period. Regarding these complications, no significant difference was found between the two groups (*p* = 0.150). In terms of the location of CMVR, Zone I lesions were associated with an increased incidence rate of complications (*p* = 0.018). Rhegmatogenous retinal detachment (RRD) was the most common and severe complication, occurring in 14 eyes (20.9%) with CMVR, mostly during the healing phase. A 25-gauge pars plana vitrectomy (PPV) procedure combined with silicone oil tamponade was performed. A 21-year-old male had bilateral CMVR; after this condition was resolved, one eye developed repeated RRD, and the other developed IRU. PPV and silicone oil tamponade were performed. Unfortunately, CMVR relapse occurred even after the eye had been filled with silicone oil in the vitreous cavity; this eye had received a total of 10 IVIs of ganciclovir 0.4 mg by the 12-month visit (Fig. 1). The patient showed resistance to multiple antiviral drugs, resulting in blindness in the eye with RRD and total vision loss in the eye with IRU.

IRU developed in 9 eyes (13.4%) of 9 patients after the resolution of CMVR. All of these patients had stable CD4^+^ T-lymphocyte counts above 200 cells/µL for more than 3 months during follow-up. IRU was characterized as 1 + to 2 + inflammation in the anterior chamber or vitreous, anterior synechia, cataract formation, vitreous opacities, cystoid macular oedema (CME) and epiretinal membrane formation. Topical corticosteroid eyedrops were used for those eyes. Four eyes with CME and vitreous opacities recovered without complications after one intravitreal triamcinolone injection each, but 5 eyes progressed to blindness because they were not treated in time or because they developed glaucoma.

Six eyes (8.9%) of 6 patients had optic atrophy secondary to glaucoma or CMVR lesions involving the optic nerve. Three eyes in the cohort had poorly controlled glaucoma because of anterior synechia. Glaucoma treatment was provided, but the vision loss was irreversible. Other complications included vitreous haemorrhage in 4 eyes (5.9%) and CMVR relapse in 2 eyes (3.0%). No evidence was found for CMVR relapse in fellow eyes or newly developed CMV end-organ disease during follow-up. A transient increase in IOP occurred universally after IVI. No serious adverse events, such as high IOP, retinal detachment, vitreous haemorrhage, or endophthalmitis, occurred in association with IVI.

## Discussion

In this study, we found that IVI of either low- or intermediate-dose ganciclovir, when combined with systemic ganciclovir therapy, produced favourable visual outcomes for HIV-positive patients with CMVR. However, these outcomes were not maintained at 12 months. Statistical analysis showed that the localization of CMVR in Zone I not only worsened the visual prognosis but also affected the number of IVIs needed. Increasing the concentration of ganciclovir within a certain range did not reduce the number of IVIs. Additionally, the number of IVIs and the rate of complications were significantly lower for lesions in Zone II than for those in Zone I.

Several studies have reported favourable clinical outcomes achieved by using various doses of ganciclovir in AIDS patients with CMVR [7, 9, 14, 17]. Our intravitreal treatment regimen was effective in healing CMVR lesions; the objective response rate was 100%, and 76.1% of eyes showed an improved or stable BCVA. Both the low-dose and intermediate-dose groups showed significant improvements in BCVA after intravitreal treatment. This is consistent with other reports; for example, Xie et al. [9] treated AIDS-related CMVR with intravitreal ganciclovir 3 mg 2 times per week, and all patients showed improved BCVA after treatment (a 100% response rate), with 44% of eyes clinically cured. Teoh et al. [17] reported an 88% response rate, with 80% of eyes achieving stable or improved BCVA after intravitreal treatment for AIDS-related CMVR. However, a gradual loss of vision was observed because macular or optic atrophy continued even if the lesion became inactive, which caused the BCVA at the final visit to show no significant improvement from baseline following either low-dose or intermediate-dose treatment. Similar disappointing results had been reported in previous studies, in which both systemic and intravitreal ganciclovir therapy were applied. Shoeibi et al. [18]. reported a decrease in the mean logMAR BCVA by 0.57 to 0.69 from baseline to the final visit despite appropriate and aggressive treatment. Previous studies have analysed the prognostic factors for CMVR in HIV-negative patients; these studies demonstrated that the severity of clinical features at the time of diagnosis, the proximity of the lesion to the posterior pole, and the extent of the CMV lesion were significantly related to poor visual outcomes [19, 20]. In HIV-infected patients with CMVR, peripheral blood CMV DNA levels of > 6,390 copies/mL were associated with a poor prognosis [21]. However, we failed to measure the serum CMV DNA levels. In our study, Zone I lesions involving the macula or optic nerve were associated with severe vision loss (*p* < 0.001). Continuing macular and optic atrophy occurred only in Zone I, which may explain such findings. Moreover, half of the eyes in our study had a poor BCVA at baseline. Therefore, we speculated that baseline BCVA was responsible in part for the poor visual outcomes of CMVR in AIDS patients.

Previous studies demonstrated that the number of injections needed was positively associated with the initial aqueous CMV DNA load [20]. We expected that increasing the injection dosage of ganciclovir would reduce the number of IVIs. However, the results that we observed in a clinical setting were not as expected; when the injection dosage was increased 2.5-fold, the mean number of IVIs did not decrease. A previous study increased the injection dosage from 1 mg to 6 mg (a 6-fold increase) in HIV-negative patients with CMVR, and the mean total number of IVs was significantly reduced [8]. We observed that CD4^+^ T-lymphocyte counts continuously increased after systemic and intravitreal antiviral therapy, which corresponded to atrophy of fundus lesions. The increased CD4^+^ T-lymphocyte counts represent clinical evidence of CMV-protective immunity [22]. Moreover, we found that lesions in Zone I required more injections than lesions in other zones (*p* = 0.036). This was slightly different from the results reported by Qian ZY et al. [20]; in their study, the location of CMVR was not associated with the number of IVIs. The difference may be related to the different characteristics of the participants (HIV-positive patients in our study and HIV-negative patients in the study conducted by Qian ZY et al.).

Zone I lesions were associated with the highest number of complications at the 12-month visit. RRD and IRU were the two major complications (20.9% and 13.4%, respectively). The development of RRD was a consequence of full-thickness retinal necrosis resulting in multiple holes and retinal breaks, which were usually in the atrophic area of the retina. However, we found that even with vitrectomy and silicone oil tamponade, postoperative vision was often limited in CMVR patients with RRD. Some studies have recommended prophylactic laser treatment or early vitrectomy to reduce the risk of retinal detachment [23]. However, we did not conduct laser treatment or early vitrectomy. Given the high incidence of retinal detachment (20.9%), performing early vitrectomy may be necessary in cases of extensive CMVR involvement. IRU is an ocular manifestation of immune reconstitution inflammatory syndrome (IRIS), which occurs during immune recovery driven by ART. IRU develops in 15–25% of HIV-positive patients with CMVR [24]. The overall incidence of IRU in our study was 13.4%, which was slightly lower than previously reported rates. Notably, all cases of IRU in this study were found in patients with bilateral CMVR. This finding was consistent with those in a previous study, and these results suggested that larger lesions and bilateral CMVR were related to the development of IRU in CMVR patients [18]. In addition, other risk factors for IRU in previous reports included elevated CD4 counts, regressed CMVR, and use of intravitreous injections of cidofovir [25].

One of the limitations of this study is that we measured the aqueous CMV DNA load only at baseline, which resulted in a lack of quantitative indicators for discontinuing the IVIs. The injections were stopped based only on the change in the fundus, which introduced subjectivity and thus created the potential for bias.

## Conclusions

Weekly IVI of either 0.4 mg or 1.0 mg ganciclovir effectively produced favourable visual outcomes in patients with AIDS-related CMVR. However, irreversible vision loss continued to occur in the long term despite aggressive antiviral treatment. Within a certain range, the dosage of ganciclovir did not affect the number of IVIs needed. It is important to distinguish the location of the lesion in CMVR patients; a CMVR lesion in Zone I may portend a poor visual outcome, an increased number of IVIs, and an elevated rate of CMVR-related complications.

## Data Availability

The datasets used and/or analysed during the current study are available from the corresponding author on reasonable request.
